# Validating Well-Functioning Hepatic Organoids for Toxicity Evaluation

**DOI:** 10.3390/toxics12050371

**Published:** 2024-05-17

**Authors:** Seo Yoon Choi, Tae Hee Kim, Min Jeong Kim, Seon Ju Mun, Tae Sung Kim, Ki Kyung Jung, Il Ung Oh, Jae Ho Oh, Myung Jin Son, Jin Hee Lee

**Affiliations:** 1 Division of Toxicological Research, National Institute of Food and Drug Safety Evaluation, Ministry of Food and Drug Safety, Cheongju 28159, Republic of Korea; sugar0819@korea.kr (S.Y.C.); th0919@korea.kr (T.H.K.); jeong8328@korea.kr (M.J.K.); ktsung@korea.kr (T.S.K.); ollong@korea.kr (I.U.O.); chopin68@korea.kr (J.H.O.); 2 Department of Orthopaedic Surgery, David Geffen School of Medicine, University of California Los Angeles, Los Angeles, CA 90095, USA; 3 Stem Cell Convergence Research Center, Korea Research Institute of Bioscience and Biotechnology (KRIBB), Daejeon 34141, Republic of Korea; sjmoon@kribb.re.kr; 4 Division of Pharmacological Drug Research, National Institute of Food and Drug Safety Evaluation, Ministry of Food and Drug Safety, Cheongju 28159, Republic of Korea; kikyung@korea.kr; 5 Department of Functional Genomics, Korea University of Science & Technology (UST), Daejeon 34113, Republic of Korea

**Keywords:** liver organoid, liver toxicity test, 3D culture, alternative test

## Abstract

“Organoids”, three-dimensional self-organized organ-like miniature tissues, are proposed as intermediary models that bridge the gap between animal and human studies in drug development. Despite recent advancements in organoid model development, studies on toxicity using these models are limited. Therefore, in this study, we aimed to analyze the functionality and gene expression of pre- and post-differentiated human hepatic organoids derived from induced pluripotent stem cells and utilize them for toxicity assessment. First, we confirmed the functional similarity of this hepatic organoid model to the human liver through various functional assessments, such as glycogen storage, albumin and bile acid secretion, and cytochrome P450 (CYP) activity. Subsequently, utilizing these functionally validated hepatic organoids, we conducted toxicity evaluations with three hepatotoxic substances (ketoconazole, troglitazone, and tolcapone), which are well known for causing drug-induced liver injury, and three non-hepatotoxic substances (sucrose, ascorbic acid, and biotin). The organoids effectively distinguished between the toxicity levels of substances with and without hepatic toxicity. We demonstrated the potential of hepatic organoids with validated functionalities and genetic characteristics as promising models for toxicity evaluation by analyzing toxicological changes occurring in hepatoxic drug-treated organoids.

## 1. Introduction

Drug toxicity evaluation is a pivotal phase in drug development, ensuring human safety and detecting potential toxic effects prior to clinical trials [1]. Traditionally reliant on animal experimentation, toxicity assessments face challenges owing to interspecies physiological variations and the global advocacy for the three Rs principle (reduction, refinement, and replacement) [2]. Consequently, alternative methods have emerged, including computer-aided drug design software and non-animal models like cell-based assays, microphysiological systems, and computer simulations [3]. Among these alternatives, the organoid platform has garnered considerable interest for faithfully replicating human organ complexity. Organoids, derived from induced pluripotent or adult stem cells, self-organize in three-dimensional (3D) cultures, closely mirroring human organ structures and functions [4]. Bridging the gap between two-dimensional (2D) cell cultures and in vivo models, organoids offer advantages such as structural and functional resemblance to primary tissues and enhanced genome editing capabilities [5].

While 3D cultured cell aggregates, known as spheroids, are structurally closer to in vivo conditions than conventional 2D cell cultures and are simpler to generate, making them convenient for drug screening, they often possess less intricate structures than self-organized organoids [6]. Additionally, as the duration of suspension culture increases, nutrient and oxygen diffusion into the cell aggregate becomes challenging, while waste removal from the interior becomes limited, often leading to the development of central necrosis [7]. In contrast, self-organized 3D organoids, often cultured in matrix support within a medium supplemented with growth factors and cytokines, possess a more complex structure and cell composition than spheroids. These attributes indicate the potential suitability of organoids for advanced applications across toxicology, OMICs, gene editing, drug development, and precision medicine [8,9]. Notably, organoids from multiple organs, including the gut, stomach, lungs, kidneys, liver, pancreas, and brain, have been developed, leading to active research on diverse organ systems [8].

The liver, the most metabolically active tissue per unit weight, plays a crucial role in metabolizing most drugs [10]. Drug-induced liver injury (DILI) presents a significant challenge in drug development owing to its dose-dependent toxicity and the occurrence of idiosyncratic liver toxicity. In vitro cultured primary human liver cells closely mimic the in vivo liver with minimal reprogramming and differentiation requirements, rendering them valuable for drug screening [11]. However, they have shorter culture timespan and lack reproducibility. Liver-on-a-Chip, based on microfluidic technology, can also serve as a promising model that closely mimics the structure and physiology of the liver, even controlling the concentration of oxygen and nutrients [12]. However, this model may not be suitable for high throughput analysis and could pose challenges for drug screening due to the need for diverse fabrication. Therefore, liver organoids are considered highly suitable models for studying hepatotoxicity owing to their cellular and functional similarity to the liver and suitability as a drug screening model. Notably, toxic metabolites can impact enzymes, nuclei, and transporters in liver cells, allowing for the observation of changes in organoids treated with toxic substances to understand their effects on liver cells [6].

Despite recent advancements in organoid models, limited studies have delved into their toxicity. DILI stands out as the leading cause of regulatory actions, including pharmaceutical market withdrawals [13]. There is increasing interest in advancing liver organoid models for more effective prediction of drug toxicity, aiming to address the limitations of 2D cell cultures and in vivo models [14]. Previously, we developed liver organoids and identified various cellular compositions of liver cells, including hepatocytes, cholangiocytes, hepatic stellate cells, and immune cells within the liver organoids as determined by single-cell analysis [14,15]. In this study, we aimed to evaluate the functionality of these liver organoid models and to determine whether these liver organoids are suitable for toxicity testing by subjecting them to DILI substances. After confirming functional similarity, our results indicated that our established organoids could distinguish toxicity levels between DILI and non-hepatotoxic substances, indicating their suitability for assessing liver toxicity.

## 2. Materials and Methods

### 2.1. Organoid Culture and Differentiation

The iPSCs used for organoid generation were derived from normal human skin fibroblasts (catalog number: CRL-2097), obtained from the American Type Culture Collection (ATCC, Manassas, VA, USA). The organoid generation methods were described in detail in our previous study [14]. For scalable generation, cells in the mature hepatocyte state, formed in a 3D structure, were detached using a blade and then embedded into Matrigel (catalog number: 354234; Corning Inc., Corning, NY, USA) with hepatic medium (HM; composition detailed in Table 1) containing 10 μM Y-27632 (catalog number: 1254; Tocris Bioscience, Bristol, UK) for 3 days. Three-dimensional liver organoids formed within 3–5 days, and the generated organoids were replenished with the HM medium every 2–3 days. Every 7 days, organoids underwent mechanical passage, which involved being cut into small pieces with a surgical blade or tissue chopper (model number: TC752; Mcilwain; Ted Pella Inc., Redding, CA, USA), following a wash with cold phosphate-buffered saline (PBS) to remove Matrigel. Cells were then resuspended in Matrigel in a ratio of 1:3–1:5. For cryopreservation, organoids were mixed with mFreSR (catalog number: 05855; StemCell Technologies, Vancouver, BC, Canada), frozen, and thawed as per established protocols [14]. Post-thawing, Y-27632 was added to the medium and incubated for 3 d. Differentiation of hepatic organoids involved initial incubation in expansion medium (EM) for 2–3 d followed by differentiation medium (DM) for an additional 8 d. Table 2 and Table 3 detail the compositions of the expansion and differentiation media, respectively.

### 2.2. Quantitative PCR

To assess the genetic characteristics of the differentiated organoids, total RNA was isolated using TRIzol reagent (catalog number: 1596018; Invitrogen, Waltham, MA, USA) following the manufacturer’s protocol. RNA was reverse-transcribed into cDNA using a 96-well Thermal Cycler (Veriti; Thermo Fisher Scientific, Waltham, MA, USA) and Topscript RT Dry Mix (catalog number: RT200; Enzynomics, Daejeon, Republic of Korea). Quantitative analysis of gene expression levels in the cDNA samples was conducted using an AB 7500 Fast Real-time PCR (7500 Fast; Thermo Fisher Scientific) and SYBR Green PCR Master Mix (catalog number: 4309155; Thermo Fisher Scientific). The primers used are listed in Table 4.

### 2.3. Immunofluorescence Assay

The organoids were seeded onto a Nunc™ Lab-Tek™ II Chamber Slide™ System (catalog number: 154461PK; Thermo Fisher Scientific) and differentiated. Afterward, cells underwent 2–3 washes with Dulbecco’s phosphate-buffered saline (DPBS) and were fixed in 4% paraformaldehyde (PFA) at 4 °C for 1 h. Following fixation, organoids were washed three times using phosphate-buffered saline with 0.1% Tween^®^ 20 (PBS-T). To permeabilize cells, they were treated with 0.1% Triton X-100 for 15 min. This step was followed by three PBS-T washes. Cells were then incubated with 4% bovine serum albumin at room temperature (15–25 °C) for 1 h. Subsequently, anti-albumin (ALB) antibody (catalog number: A80-129A; Bethyl Laboratories, Inc., Montgomery, TX, USA) was applied and incubated overnight at 4 °C. After three PBS-T washes, Alexa Fluor 488 antibody (catalog number: A11055; Life Technologies, Carlsbad, CA, USA) was applied at room temperature for 1 h in the dark, followed by three PBS-T washes. After completion, the slide glass mold was removed per the manufacturer’s instructions. DAPI mounting solution (20 μL per well) was applied and incubated at room temperature for 5 min. Fluorescent images were then acquired using a Zeiss LSM 700 confocal microscope (Carl Zeiss, Tübingen, Germany).

### 2.4. Tissue Clearing and Immunostaining

Samples collected pre- (HM) and post (DM)-differentiation underwent 2–3 DPBS washes. Subsequently, cells were fixed in 4% PFA at 4 °C for 1 h. Tissue clearing was achieved using a Binaree^®^ Tissue Clearing™ Kit (catalog number: HRTC-012; Binaree, Daegu, Republic of Korea). Briefly, tissues were immersed in a fixing solution for 24 h, followed by immersion in a tissue-clearing solution. For permeabilization, tissues were incubated with 0.2% Triton X-100 at 37 °C for 24 h in an incubator shaker. Samples were then subjected to a 4 d incubation at 37 °C with the primary antibody (catalog number: 3113s; Cell Signaling Technology, Danvers, MA, USA), followed by washing and a 2-day incubation at 37 °C with the secondary antibody (catalog number: A32740; Life Technologies). DAPI nuclear staining was performed at a 1:100 ratio and the samples were then incubated at 4 °C for 2 h, followed by three washes. Finally, samples were incubated in a Binaree^®^ Tissue Clearing™ Mounting Solution (catalog number: HRMO-006; Binaree) at 37 °C for 1 d. Mounted samples were stored in the dark at room temperature until imaging.

### 2.5. Liver-Specific Gene Expression Panel Analysis

Liver similarity analysis was conducted as previously described [16,17]. The liver-specific gene expression panel (LiGEP) algorithm was quantified using the fragments per kilobase of transcript per million mapped reads (FPKM) value. The LiGEP score was calculated by extracting the expression values of the 98 genes from the LiGEP panel. To quantify the percentage of LiGEP, we confirmed the liver-specific gene expression results using the FPKM algorithm, assessing the similarity to liver samples using the Web-based Similarity Analysis System algorithm.

### 2.6. RNA Sequencing of HM and DM Samples

RNA sequencing was performed using Macrogen: www.macrogen.co.kr (accessed on 14 November 2022). For RNA sequencing, total RNA was isolated using the TRIzol reagent (catalog number: 1596018; Invitrogen), following the manufacturer’s instructions. For sequencing, a minimum of 1 μg of RNA per sample was required. We used approximately 100–150 organoids per sample to obtain this amount of RNA. We confirmed RNA integrity using a 2100 Bioanalyzer (Agilent Technologies, Santa Clara, CA, USA) and performed RNA sequencing using high-quality total RNA samples (the RNA integrity number ratio of all samples was 10). RNA sequencing was performed using a NovaSeq 6000 platform (Illumina, CA, USA). Raw reads were checked for quality using FastQC v0.11.7. Processed reads were aligned to the human reference genome (GRCh38) using HISAT2 software v2.1.0. To compare the organoid transcriptome with that of the human liver tissue, we downloaded a gene expression profiling dataset (GSE159720) from the GEO database. In this study, we used the BA recipient liver and three normal liver allograft samples (GSM4838478, GSM4838479, and GSM4838480) from GSE159720. Differentially expressed genes (DEGs) were identified using DESeq2. For statistical analysis, the nbinomWald test using DESeq2 and the fold change for each comparison combination were used. Next, the similarity of each gene was grouped through a hierarchical cluster analysis of the list of significant genes and visualized as a heatmap.

To further understand their potential mechanisms and functions, we examined gene expression. Gene expression levels were compared using FPKM values. Gene ontology and Kyoto Encyclopedia of Genes and Genomes (KEGG) analyses were performed. A total of 46,427 genes were mapped, and we excluded any transcripts with an FPKM count value of zero, leaving 13,916 transcripts for the DEG analysis. Gene set enrichment analysis was performed on the normalized counts generated using DESeq2. Ranked significant gene lists were then subjected to gene ontology enrichment analysis using the g: Profiler tool (http://biit.cs.ut.ee/gprofiler (accessed on 28 November 2022)) and to gene set enrichment analysis using the KEGG database (http://www.genome.jp/kegg/ (accessed on 28 November 2022)).

### 2.7. Periodic Acid–Schiff Staining

To assess glycogen storage, we performed Periodic acid–Schiff (PAS) staining using a PAS kit (catalog number: IW-3009; IHC World, Ellicott City, MD, USA) following the manufacturer’s protocol. Briefly, the organoids were fixed with 4% PFA and then dissociated from the Matrigel. Next, the fixed samples were embedded in an OCT compound (catalog number: 4583; Sakura Finetek Co19., Ltd., Tokyo, Japan) and sliced into 8 μm sections using a cryostat microtome (CM1520; Leica, Wetzlar, Germany) at a temperature of −25 °C. Frozen sections were stained with PAS and Mayer’s hematoxylin solution (catalog number: IW-3009C; IHC World). Images of the PAS-stained samples were acquired using an inverted microscope (BX53; Olympus, Tokyo, Japan).

### 2.8. Indocyanine Green Uptake and Release

To assess the uptake and release of indocyanine green (ICG), the organoids were washed with cold PBS to remove the Matrigel and then incubated with 1 mg/mL ICG (catalog number: I2633; Sigma-Aldrich, St. Louis, MO, USA) for 15 min at 37 °C in 5% CO_2_. Images of ICG uptake were captured using a microscope (DM 3000; Leica). The organoids were then gently washed thrice with PBS, and a fresh medium was added. After 1 h of incubation at 37 °C in 5% CO_2_, ICG release images were captured under a microscope.

### 2.9. Functional Polarization Assay

To conduct the functional polarization assay, the organoids were taken out of the Matrigel and incubated with culture media that contained 10 μg/mL 5-carboxy-2′,7′-dichlorofluorescein diacetate (CDFDA) (catalog number: 21884; Sigma-Aldrich) and 1 μg/mL Hoechst 33342 (catalog number: 62249; Invitrogen) for 30 min at 37 °C in 5% CO_2_. Organoids were gently washed twice with cold PBS containing calcium and magnesium. Following this, the culture medium was added and fluorescence images were obtained using a confocal microscope (LSM 800, Carl Zeiss) at 37 °C in 5% CO_2_.

### 2.10. ALB Secretion Quantification

To measure the amount of secreted ALB, the medium was collected 24 h after changing and analyzed using a human ALB ELISA kit (catalog number: E80-129; Bethyl Laboratories), per the manufacturer’s instructions. The absorbance was measured using a microplate reader (S1LFA; BioTek Instruments, Inc., Winooski, VT, USA), and the data were adjusted for the number of cells.

### 2.11. Alpha-1 Antitrypsin Assay

To quantify the level of alpha-1 antitrypsin (AAT), the culture medium was collected 24 h after replacement with fresh medium and analyzed using a human AAT ELISA quantitation kit (catalog number: EA5101-8; Assaypro, St. Charles, MO, USA), following the manufacturer’s instructions. The absorbance was measured using a microplate reader (S1LFA; BioTek Instruments, Inc.), and the results were normalized to the cell number.

### 2.12. Analysis of Total Bile Acids

To quantify bile acid levels, the organoids were lysed by sonication with cold PBS, and the organoid lysate was separated by centrifuging at 12,000× *g* for 20 min at 4 °C. Total bile acids in the organoid supernatant were quantified using a bile acid assay kit (catalog number: ab239702; Abcam, Cambridge, UK) following the manufacturer’s instructions. The absorbance was measured using a microplate reader at a wavelength of 450 nm, and the data were normalized to the cell number. The data were analyzed per the manufacturer’s instructions.

### 2.13. Analysis of CYP3A4 Enzyme Activity

CYP enzyme activity was measured using a P450-Glo Assay Kit (catalog number: V9002; Promega, Madison, WI, USA). HM-cultured and DM-cultured organoids in a 24-well plate were treated with 10 μM nifedipine (catalog number: N7634; Sigma-Aldrich) for 48 h. After washing the organoids with basal media, they were treated with Luciferin-IPA (luminogenic CYP substrate) at a concentration of 3 μM in 300 μL per well and incubated for 3 h. In total, 25 μL of the mixture was transferred to a white opaque 96-well plate, mixed with 25 μL of Luciferin detection reagent, and incubated in the dark at room temperature on a shaker for 20 min. Luciferase activity was measured using a luminometer (catalog number: GM2000; Promega) with an integration time set to 1 s. Data were normalized by the cell number.

### 2.14. Analysis of Toxicity Test

Liver organoid differentiation was performed as previously described [14]. We first confirmed the functionality of the differentiated liver organoids by measuring CYP3A4 expression at the mRNA level and ALB secretion. The differentiated organoids were seeded in a U-shaped 96-well plate (catalog number: 34296; SPL Life Sciences, Gyeonggi-do, Republic of Korea) and stabilized for 48 h. We then treated the organoids with either DILI or non-hepatotoxic substances daily for 5 days. The drugs were prepared in Dimethyl Sulfoxide (DMSO) (catalog number: sc-358801; Santa Cruz Biotech.) or water (catalog number: 10977-015; Invitrogen) to prepare stocks, which were then diluted in media according to the desired concentrations. The DMSO percentage in all experimental groups was kept constant and did not exceed 1%. On the last day, the supernatant was collected and preserved at −80 °C for subsequent albumin analysis. After removing all supernatant, EZ-Cytox reagent (catalog number: EZ-1000; DoGenBio, Seoul, Republic of Korea) was added to each well according to the manufacturer’s instructions to measure the remaining cell viability. The absorbance at 450 nm was then measured using a microplate reader to determine the cell viability of the substance-treated cells. The cell viability was determined relative to the absorbance of the control. Data are presented as a percentage of cell viability (% of control), calculated as follows:

$$\mathrm{Cell}\;\mathrm{Viability}\;\left(\%\right)=\frac{\mathrm{Sample}-\mathrm{Blank}}{\mathrm{Control}-\mathrm{Blank}}\times 100$$

The concentration at which each substance inhibited 50% of liver organoid growth (TC_50_) was determined by plotting the logarithm of substance concentration on the *x*-axis and the percentage of cell viability on the *y*-axis. To generate a curve fitting these data points, the following equation was used: log (inhibitor) vs. normalized response variable slope.

*Y* = 100/[1 + 10^((logTC50 − *x*) × Hill Slope)^]

### 2.15. Gene Ontology Functional Enrichment Analysis of Drug-Treated Samples

Total RNA concentration was calculated by Quant-IT RiboGreen (catalog number: R11490; Invitrogen). To assess the integrity of the total RNA, samples were run on a TapeStation RNA screentape (catalog number: 5067-5576; Agilent Technologies). Only high-quality RNA preparations, with an RIN greater than 7.0, were used for RNA library construction. A library was independently prepared with 0.5 µg of total RNA for each sample using an Illumina TruSeq Stranded Total RNA Library Prep Gold Kit (catalog number: 20020599; Illumina). The first step in the workflow involves removing the rRNA in the total RNA. Following this step, the remaining mRNA is fragmented into small pieces using divalent cations under elevated temperatures. The cleaved RNA fragments are copied into first-strand cDNA using SuperScript II reverse transcriptase (catalog number: 18064014; Invitrogen) and random primers. This is followed by second-strand cDNA synthesis using DNA Polymerase I, RNase H, and dUTP. These cDNA fragments are then put through an end repair process, the addition of a single ‘A’ base, and then ligation of the adapters. The products are then purified and enriched with PCR to create the final cDNA library. The libraries were quantified using KAPA Library Quantification kits for Illumina Sequencing platforms according to the qPCR Quantification Protocol Guide (catalog number: KK4854; KAPA BIOSYSTEMS, Wilmington, MA, USA) and qualified using the TapeStation D1000 ScreenTape (catalog number: 5067-5582; Agilent Technologies). Indexed libraries were then submitted to an Illumina NovaSeq (Illumina, Inc., San Diego, CA, USA), and paired-end (2 × 100 bp) sequencing was performed. All analyses were carried out in R v4.2.2 with specific software packages. Sequencing data were normalized using DESeq2 v1.38.3 [18] and applied for the identification of DEGs. Genes were classified as differentially expressed when their *p* value was <0.05, and they exhibited a log2 fold change greater than 2 or less than −2. A heatmap was generated using GraphPad Prism 10 (GraphPad Software ver. 10, La Jolla, CA, USA) and gene ontology (GO) was performed with clusterProfiler v4.6.2 [19], which supports statistical analysis and visualization of functional profiles for genes and gene clusters.

### 2.16. Cell Counting

Cell counting of the organoids was performed using a hemocytometer under a microscope, per the manufacturer’s protocol. In brief, we washed the organoids with basal medium and DPBS and then dissociated them into single cells using the TrypLE Express enzyme (catalog number: 120536; Gibco, Waltham, MA, USA). We then measured the number of viable cells using the trypan blue exclusion cell counting method and calculated the number of viable cells/mL in the cell suspension.

### 2.17. Statistical Analysis

Data were analyzed using GraphPad Prism 10 (GraphPad Software ver. 10). All data are expressed as mean ± standard error of the mean. ALB and AAT secretion data were compared using one-way analysis of variance with post hoc Tukey pair-wise comparisons. Student’s *t*-test was used to assess intergroup comparisons of qPCR, bile acid production, and CYP3A4 activity data. A value of *p* < 0.05 was considered statistically significant.

## 3. Results

### 3.1. Mature Characterization of the Differentiated Human Liver Organoid

**Figure 1:**
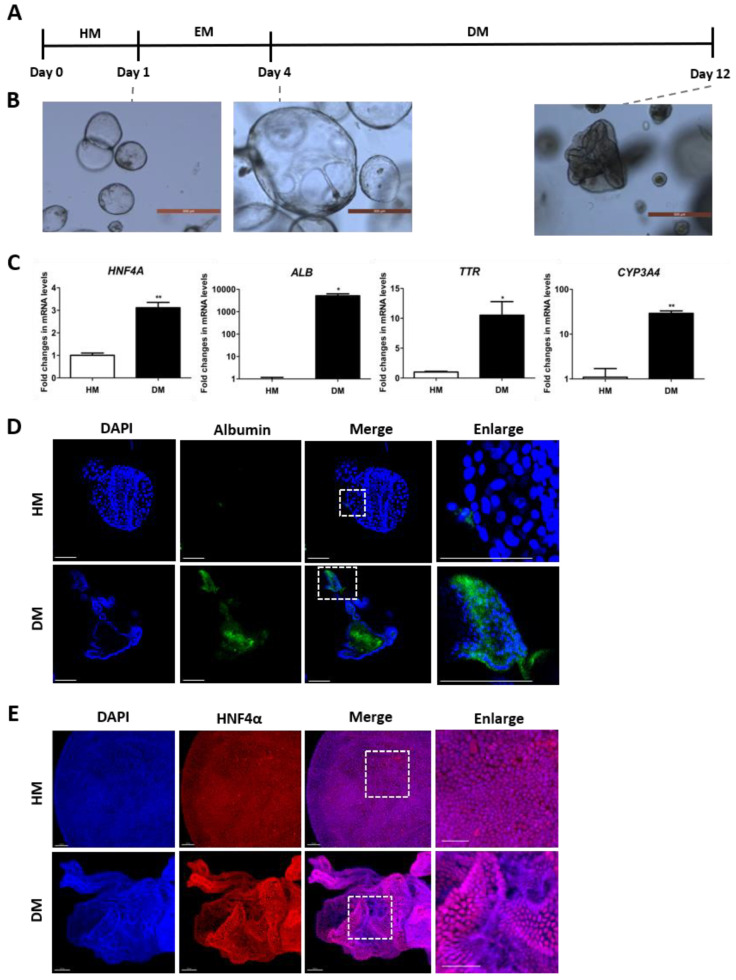
Establishment of a functional liver organoid. (**A**) Schematic representation of the liver organoid differentiation process. (**B**) Representative morphology of hepatic organoids at HM-, EM-, and DM-cultured maturation stages. Scale Bar = 500 µm. (**C**) mRNA expression of each hepatocyte marker and drug metabolism gene. The data are presented as the mean ± standard error of the mean (n = 2) and were analyzed by Student’s *t*-test, * *p* < 0.05, and ** *p* < 0.01 compared to HM. (**D**) Immunofluorescence staining of whole organoids in each differentiation stage. Immunofluorescence staining for albumin (green) and nuclei are stained with DAPI (blue). Scale Bar = 100 µm. (**E**) Representative immunostaining images of HNF4α (red) and nuclei (blue) after tissue clearing. Scale Bar = 50 µm. HM, hepatic medium; EM, expansion medium; DM, differentiation medium; HNF4α, hepatocyte nuclear factor 4-alpha; ALB, albumin; TTR, transthyretin.

To obtain scalable hepatic organoids, 3D hepatic organoids were derived from pluripotent stem cells. The procedure for organoid generation has been previously described [14,20]. For hepatic maturation, organoids were cultured in defined media (Table 1, Table 2 and Table 3). The culture conditions involved HM followed by EM for 3 d to induce hepatocyte proliferation. Subsequently, organoid differentiation was induced by culturing in DM for 8 d (Figure 1A). Representative morphological features of the culture medium are depicted in Figure 1B. HM organoids typically displayed cystic structures with clear lumens and a thin monolayer of cells. EM-cultured organoids exhibited an expanded spherical structure compared to HM-cultured organoids. DM-cultured organoids showed distinct cell differentiation morphologies, with smaller, more folded structures and darkened lumens compared to HM-cultured organoids.

Previous studies have linked ALB, hepatocyte nuclear factor 4-alpha (HNF4α), and transthyretin (TTR) with hepatocyte maturity progression [21,22]. Hence, we assessed the mRNA expression levels of mature hepatocyte markers (*HNF4α*, *ALB*, *TTR*) and the drug-metabolizing gene *CYP3A4*. In DM organoids, the expression of mature hepatocyte markers and drug metabolism genes was significantly higher compared to that in HM organoids (Figure 1C). When comparing the expression of these genes with primary human hepatocytes (PHH), hepatocyte markers such as ALB and HNF4A showed similar or higher expression levels on day 8 of differentiation. However, CYP3A4 exhibited lower expression compared to PHH (Figure S1). Further investigations into ALB and HNF4α expression levels in HM and DM organoids using immunofluorescence revealed significantly increased ALB expression in DM compared to HM within the organoid (Figure 1D). Conversely, HNF4α expression remained consistently high in both HM and DM groups (Figure 1E). Collectively, the results indicate that hepatic organoids undergo morphological changes and increased hepatocyte marker expression in mRNA and protein levels during differentiation.

### 3.2. Analysis of Transcriptome Profiles in Human Hepatic Organoids

**Figure 2:**
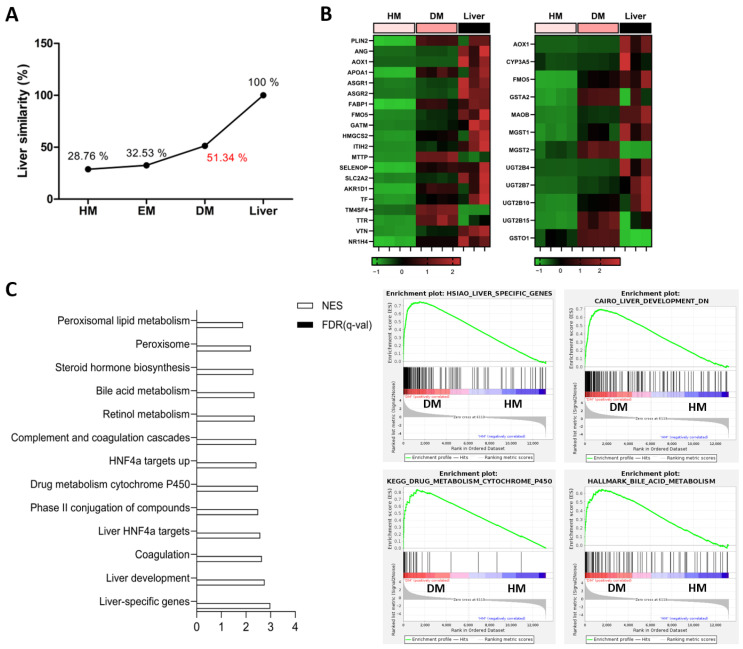
Transcriptome analysis in liver organoids. (**A**) Liver similarity analyzed using the LiGEP algorithm; HM-, EM-, and DM-cultured organoids; (**B**) Heatmap based on DEGs from liver-specific gene sets (left) and drug metabolism gene sets (right). (**C**) List of gene sets enriched in DM when compared to HM. Enrichment plot showing the top-ranked subset signatures in DM: liver-specific genes, liver development, drug metabolism cytochrome P450, bile acid metabolism. LiGEP: liver-specific gene expression panel. NES: Normalized Enrichment Score. FDR; False Discovery Rate.

We conducted RNA sequencing to assess the maturity of the organoids on day 8 of differentiation. Utilizing the LiGEP algorithm, we confirmed liver similarity and scrutinized the molecular profiles at various differentiation stages. At the pre-differentiation stage (HM), the liver similarity score was relatively low (28.76%). However, as differentiation progressed into the EM and DM stages, liver similarity increased to 32.53% and 51.34%, respectively, indicating an ascending trend towards human liver similarity (Figure 2A). Additionally, we performed differential gene expression (DEG) analysis among pre-differentiated organoids (HM), post-differentiated organoids (DM), and human liver groups using RNA sequencing data. We observed the upregulation of liver-specific and drug metabolism-related genes in the DM group. Moreover, when compared through a heatmap, the expression of these genes in the DM organoids appeared more similar to the gene expression in the human liver than that in the HM organoids. (Figure 2B). Furthermore, comparing enriched pathways in the post-differentiation to pre-differentiation states, gene sets associated with liver development and liver-specific functions were upregulated, concomitant with the activation of pathways involved in lipid, bile acid, and drug metabolism (Figure 2C). In summary, our findings suggest that not only do liver organoids mature in terms of functionality during differentiation, but they also exhibit changes in gene expression profiles.

### 3.3. Functional Evaluation of Liver Organoid

**Figure 3:**
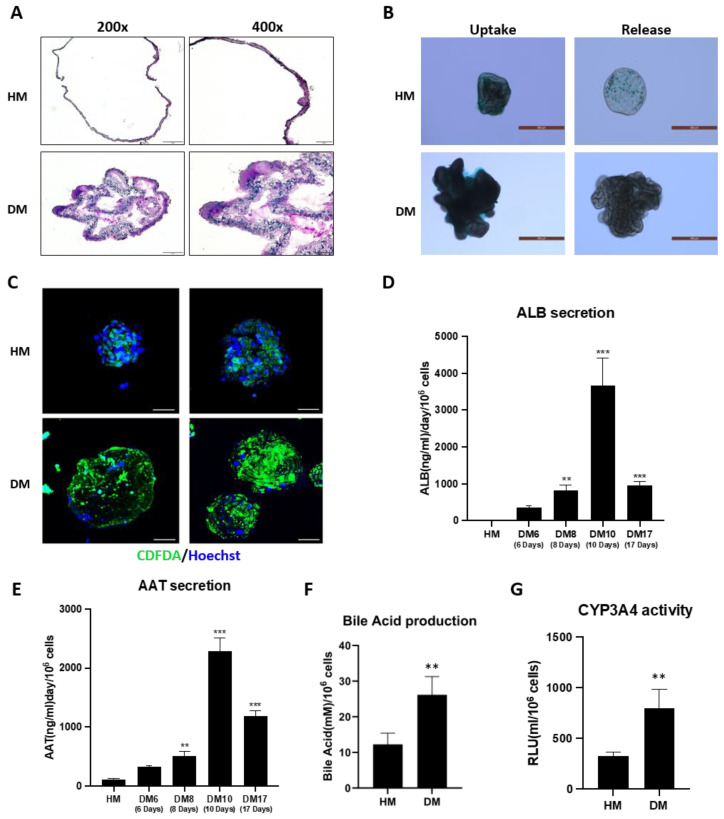
Functional evaluation of liver organoid. Representative images of HM- and DM-cultured liver organoids (**A**) stained with PAS. The 200× Scale Bar = 50 µm; 400× Scale Bar = 20 µm. (**B**) Representative images of liver organoids cultured in HM and DM, following a 15 min incubation with ICG. Scale Bar = 500 µm. (**C**) Representative fluorescence images illustrating bile canaliculi-like structures, stained with CDFDA (green) and Hoechst 33342 (blue), in organoids cultured in HM and DM. Scale Bar = 50 µm. (**D**) Quantification analysis of albumin secretion, and (**E**) AAT secretion detected by ELISA. Data are expressed as mean ± standard deviation (n = 3) and compared by one-way analysis of variance with Tukey’s post hoc to HM. ** *p* < 0.01; and *** *p* < 0.001. (**F**) Bile acid production and (**G**) Nifedipine-induced CYP3A4 enzyme activity. Data are expressed as mean ± standard deviation (n = 4) and analyzed by Student’s *t*-test. PAS, Periodic acid–Schiff; ICG, indocyanine green; CDFDA, carboxy-2′,7′-dichlorofluorescein diacetate; AAT, alpha-1 antitrypsin.

We performed various assessments of hepatic organoid function on day 8 of differentiation to verify the establishment and successful implementation of the characteristics and functionalities of the constructed organoids. The glycogen storage function of the organoids was confirmed by PAS staining (Figure 3A). The results showed that organoids cultured in HM typically exhibited a round morphology with a single layer and cystic structure. In contrast, differentiated organoids subjected to PAS staining displayed a higher degree of staining. These differentiated organoids were smaller in size but had a thicker layer compared to the organoids cultured in HM. In the ICG uptake images of liver organoids cultured in HM, ICG was absorbed by the organoids, resulting in a darker green color. In the efflux images, ICG was released, resulting in a lighter dye color. Similarly, in the ICG uptake images of differentiated organoids, ICG was absorbed into the organoids, resulting in a darker green color, whereas in the efflux images, ICG was released, resulting in a lighter dye color. This indicates that both the HM and DM groups exhibited well-implemented ICG uptake and release functions in the liver organoids (Figure 3B). After each culture stage, liver organoids were stained with CDFDA to confirm functional polarization through fluorescence observation using confocal microscopy (Figure 3C). In organoids cultured in HM, polarized bile canaliculi emitting green fluorescence were observed in some cells; however, most cells retained CDFDA fluorescence in the cytoplasm. Nuclei were stained with Hoechst 33342 and appeared blue. Most cells in the HM-cultured liver organoids were nonpolarized. In contrast, liver organoids cultured in DM exhibited stronger overall fluorescence than those cultured in HM. DM-cultured liver organoids demonstrated relatively well-observed bile canaliculi that excreted bile acids, indicating polarity acquisition in liver cells.

ALB secretion from liver organoids cultured in HM was almost negligible. However, when differentiated organoids were assayed for ALB secretion, the amount of secreted ALB significantly increased during the differentiation period. Liver organoids induced for differentiation for 6, 8, and 10 d demonstrated 355.89, 814.16, and 3669.33 ng/mL/day/10^6^ cells of ALB secretion, respectively. However, the albumin secretion did not increase indefinitely when differentiation persisted beyond a certain level over the long term. In organoids differentiated for 17 days, 960.93 ng/mL/day/10^6^ cells of albumin were secreted (Figure 3D). When the secretion of AAT was measured in the culture medium on differentiation days, 6, 8, 10, and 17, the results showed a pattern similar to that of albumin. Though the secretion increased as the differentiation period was extended, it exhibited a decrease in secretion after a certain period (Figure 3E). Furthermore, the expression of drug metabolism-related genes showed an increasing trend with differentiation, reaching a peak around day 6~8, followed by a decrease in expression thereafter (Figure S1). Samples obtained after cell lysis were used to quantify bile acid production using a colorimetric assay. Differentiated cells secreted significantly more bile acid than cells cultured in HM (Figure 3F). After treatment with CYP3A4-enzyme-inducing Nifedipine, the measured CYP activity in DM-cultured organoids was approximately 2.4 times higher compared to HM-cultured organoids (Figure 3G).

### 3.4. Toxicity Test

**Figure 4:**
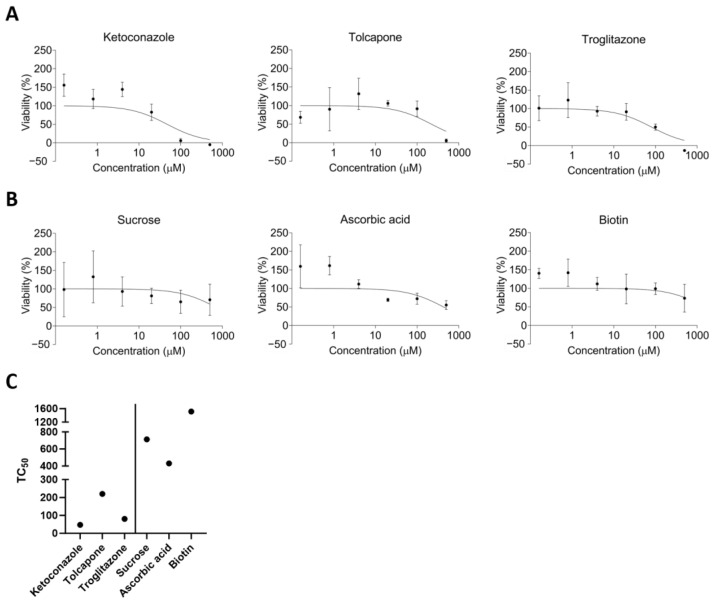
Cell viability of DILI substances and non-hepatotoxic substances. Cell viability was measured using cell-counting kit assays; (**A**) DILI substances (ketoconazole, tolcapone, and troglitazone) and (**B**) non-hepatotoxic substances (sucrose, ascorbic acid, and biotin). All substances were treated at concentrations of 500, 100, 20, 4, 0.8, and 0.16 μM. Data are expressed as mean ± standard deviation (n = 3). (**C**) TC_50_ values of the six substances. DILI, drug-induced liver injury.

To assess the suitability of liver organoids for toxicity evaluation, we conducted toxicity assessments using six substances. The substances comprised three DILI agents (ketoconazole, troglitazone, and tolcapone) and three non-hepatotoxic compounds (sucrose, ascorbic acid, and biotin) (Table 5). The classification of DILI was determined based on hepatotoxic descriptions and evidence of causality as documented in FDA-approved drug-labeling materials. Consequently, the three DILI substances were categorized as hepatotoxic (Figure 4A), while the other three substances were deemed non-hepatotoxic (Figure 4B). Toxicity was assessed to ascertain TC_50_ values (Figure 4C). Upon evaluating the results of the six substances, the three DILI agents exhibited TC_50_ values of 47.34, 220.1, and 80.5 μM, respectively, whereas the non-hepatotoxic compounds demonstrated TC_50_ values of 712.4, 430.4, and 1513 μM, respectively (Figure 4C). The liver organoids distinctly delineated the toxicities of the six substances. Overall, our research suggests that mature liver organoids exhibit significant functional and gene expression resemblances to actual livers, supporting their potential use in toxicity assessments.

### 3.5. Analysis of Albumin Secretion and Gene Ontology Enrichment in Hepatotoxic Drug-Treated Liver Organoids

**Figure 5:**
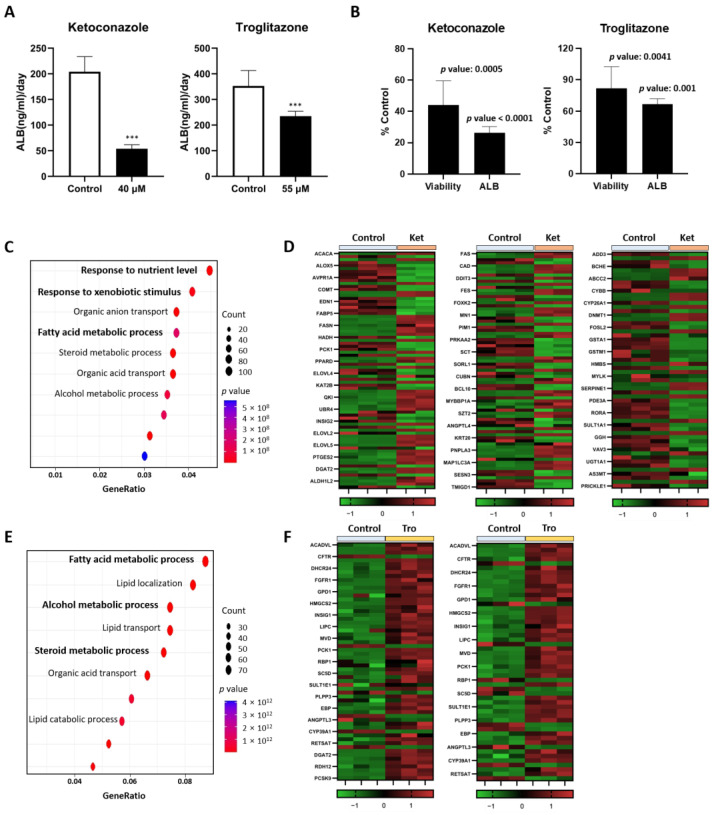
Albumin secretion and GO analysis in hepatotoxic drug-treated liver organoids. Albumin secretion (**A**) and relative viability or albumin concentration (**B**) of the cell treated with ketoconazole 40 μM and troglitazone 55 μM compared to 1% DMSO control group. The data are presented as the mean ± standard error of the mean (n = 6) and were analyzed by Student’s *t*-test, *** *p* < 0.001 compared to control. The presented *p* value means the results of a Student’s *t*-test analysis comparing the highest concentration group to the control. The GO terms using the BP algorithm dot plot (**C**) and the heatmap (**D**) of the response to the nutrient level (left), the response to xenobiotic stimulus (center), and fatty acid metabolic process (right)-related DEGs in ketoconazole-treated organoids. The GO terms dot plot using the BP algorithm (**E**) and the heatmap (**F**) of alcohol metabolic process- (left) and steroid metabolic process (right)-related DEGs in troglitazone-treated organoids. GO: gene ontology. BP: biological process. DILI: drug-induced liver injury. DEGs: differentially expressed genes.

To comprehensively examine the toxicity in liver organoids treated with hepatotoxic drugs, we treated the organoids with the TC_50_ concentrations of each drug (ketoconazole and troglitazone) obtained from toxicity tests and examined the albumin secretion and transcriptomic changes. When measuring the key indicator of liver function, albumin secretion, both the ketoconazole- and troglitazone-treated organoids exhibited a decrease in albumin secretion compared to the control group (Figure 5A). Also, when organoids were treated with 40 μM ketoconazole, viability decreased to 44.12% compared to the control, and the amount of albumin in the supernatant collected from the same experiment decreased by approximately 26.38% compared to the control (Figure 5B). In the case of troglitazone treatment at 55 μM, viability decreased to 81.86% compared to the control, and albumin secretion decreased to 66.54% (Figure 5B).

Additionally, RNA sequencing followed by GO analysis was conducted on hepatotoxic drug-treated organoids. Upon examining the top 10 statistically significant GO terms, it was observed that both drugs exhibited alterations in lipid metabolism and alcohol metabolism-related enrichment biological processes (BPs). In the ketoconazole-treated organoids, various BPs, including response to nutrient levels and response to xenobiotic stimulus, emerged, whereas troglitazone predominantly showed BPs related to lipid metabolism (Figure 5C,E). Despite alterations in lipid metabolism in both drug treatment groups, the heatmap revealed distinct gene expression patterns; while ketoconazole exhibited decreased expression of genes associated with fatty acid metabolism compared to the control, troglitazone displayed an overexpression pattern (Figure 5D,F). In conclusion, it can be observed that liver organoids demonstrate significant toxicity responses not only in terms of cell viability but also in terms of liver function and gene expression.

## 4. Discussion

In this study, we conducted various functional assessments and gene expression analyses to verify the similarity between differentiated human liver organoids and the human liver. Additionally, we assessed the toxicity of six substances associated with DILI. Overall, our liver organoids not only faithfully replicated human liver functions but also showed promise as a toxicity evaluation model by distinguishing the toxicity of the hepatotoxic substances.

HNF4α is a nuclear receptor that regulates metabolism, cell junctions, differentiation, and proliferation in the liver. TTR is a tetrameric transport protein synthesized in the liver [23]. Albumin, synthesized by liver hepatocytes, is the most abundant circulating protein in plasma. CYP3A4 is the largest member of the CYP3A subfamily and accounts for 30–60% of the total CYP450 in the adult liver. The genes encoding these proteins are specific markers for the liver and are necessary for liver cell function. When comparing the expression levels of these genes before and after the differentiation of liver organoids, their expression was significantly increased after differentiation. Furthermore, the increase in the expression of these genes suggests the possibility of an increase in the production of their encoded proteins, which can influence cell function. Examining the gene expression of 20-day differentiated liver organoids and PHHs revealed that genes associated with drug metabolism peaked in expression around days 6 to 8, followed by a subsequent decline. Among drug transporter genes, MDR1 demonstrates increased expression with differentiation, whereas MRP2 and NTCP show a decrease at day 6 followed by a gradual increase. These findings suggest that prolonged differentiation does not necessarily enhance the functionality of liver organoids. Thus, further research is warranted to identify the most functionally optimal differentiation timepoint.

In the present study, ALB secretion, a highly sensitive indicator of hepatocyte differentiation, was utilized to confirm the successful differentiation of organoids. In 2D primary human hepatocytes, approximately 1500 ng/mL/day/10^6^ ALB cells are produced [14]. Thus, on day 8 of differentiation, when ALB levels resembled those in 2D primary human hepatocytes, we conducted further functional assessments. When examining the LiGEP results on day 8 of differentiation, we observed a 51.34% similarity to the actual human liver, which is higher than that reported for 2D cells using the same analysis method [14,24]. The upregulation of liver-specific genes, drug metabolism genes, and gene expression changes resembling actual liver cells support the utilization of liver organoids for drug metabolism evaluation, ensuring reproducible liver-like reactions. Moreover, when comparing HM and DM, we noted a significant increase in ALB secretion and similar values in the DM. These findings were consistent with the RNA sequencing results, indicating the acquisition of specific hepatocellular characteristics during differentiation.

Despite its vital role in drug detoxification, current in vitro and in vivo methods fall short of fully replicating human liver physiology owing to chromosomal abnormalities, functional immaturity, and interspecies variations [16]. To address this gap, we established a liver organoid model that mimicked the functional and gene expression aspects of the human liver. In general, organoid models are cultured in a 3D system, rendering them structurally, genetically, and cellularly more similar to human organs than conventional 2D cell models [25]. While traditional 2D cell models are comparatively simpler, easier to maintain, and less costly, they fall short of adequately recapitulating human physiological aspects [4]. Furthermore, owing to their 2D nature, 2D cell models not only fail to replicate the 3D tissue environment but also face challenges in simulating interactions between the cellular and extracellular environments and interactions among cells [26]. Conversely, animal models can replicate the intricate structure and function of tissues and organs in living organisms [4]. For example, it is challenging to observe the intestinal and microbiome-associated metabolism after absorption in the intestine and transit to the liver using organoid models. Moreover, the use of such models is accompanied by ethical concerns, limitations in genetic manipulation, and higher costs [4]. Over the past three decades, a multitude of studies, encompassing mechanistic research into diseases, have been conducted utilizing animal models and 2D cancer cell lines, leading to an exponential accumulation of knowledge [4]. With recent technological advancements, organoids mimicking human organ systems have emerged, bridging the gap between the simplicity of 2D cell models and the biological resemblance of animal models.

The application of organoid models has garnered significant attention in the field of biomedicine in recent years. Human 3D organoid models, which closely resemble primary tissues through maintenance and differentiation, hold great potential for applications in studying human physiology and developmental stages. Additionally, owing to their similarities to the original organs in terms of structure and genetic information, organoid models present promising tools for biomedical research and preclinical drug testing [27]. Consequently, organoid models find application in various research areas, including human physiology, disease modeling, toxicity assessment, precision medicine, and gene editing [8]. The liver, a major organ in our body that performs various major biological functions, such as metabolism and detoxification, was first created as an organoid using rat hepatocytes in 2001 [27,28]. Overcoming initial challenges in long-term culture and achieving structural and functional characteristics, recent advancements have led to the development of liver organoid models capable of bile duct formation and bile acid secretion, as well as disease modeling, such as troglitazone-induced cholestasis [14,29]. These liver organoid technologies hold promise for applications in organogenesis modeling, liver transplantation, and drug screening [27].

Aligned with this trend, our paper presents a liver organoid model designed for preclinical hepatotoxicity screening. Using this organoid model, we conducted toxicity evaluations on a total of six substances, including three drugs—ketoconazole, troglitazone, and tolcapone—initially approved by the FDA but later withdrawn due to liver toxicity, and three non-hepatotoxic substances—sucrose, ascorbic acid, and biotin—found in the SCREEN-WELL^®^ Hepatotoxicity library (catalog number: BML-2851; Enzo Life Sciences, Farmingdale, NY, USA). In studies assessing dose–response toxicity, TC_50_, which is commonly used, indicates stronger toxicity with lower values. Recent research has utilized TC_50_ curves and values to evaluate toxicity using organoids. In the present study, TC_50_ values were adopted as toxicity indicators [14,30,31,32], and our experimental results on liver organoids revealed that TC_50_ curves and values significantly differed depending on the DILI of the substances. In other studies, the TC_50_ of hepatic organoids was similar to or lower than that measured in other in vitro models, such as cryopreserved human hepatocytes or HepaRG [33,34,35], and the cell viability curve exhibited a more gradual decrease. Considering that 3D structures typically yield higher TC_50_ values than 2D cells, our hepatic organoid model demonstrates a higher sensitivity.

To explore additional toxicity indicators besides cell viability in organoids, we examined albumin secretion and transcriptomic changes for the two drugs (ketoconazole and troglitazone) with lower TC_50_ values among the three hepatotoxic drugs tested, including ketoconazole, troglitazone, and tolcapone. Since treating organoids with drugs above their TC_50_ might not yield the sufficient RNA quantity required for RNA sequencing, we conducted measurements of albumin secretion and transcriptomics analysis in organoids treated up to TC_50_. The greater decrease in albumin secretion compared to viability reduction in the drug-treated group versus the control (1% DMSO) suggests that, along with the decrease in cell viability due to the drug, there is also a concurrent decline in organoid function. Thus, this indicates the potential of albumin secretion as a more sensitive toxicity endpoint than mere cell death. When examining the enrichment of biological processes, response to nutrient levels emerged as the top-ranking change in ketoconazole treatment. This is presumed to occur due to severe toxicity, leading to the breakdown of metabolic mechanisms and thus hindering proper mechanisms for nutrient responses. Additionally, lipid metabolism and alcohol metabolism, which are related to liver function, were among the top-ranked processes for both drugs. This suggests that hepatotoxic drugs may induce changes not only in cell survival but also in functional aspects. Furthermore, it is highly likely that underlying mechanisms also exert significant influence. Since such metabolic changes may not be easily discernible phenotypically, we aim to utilize these data as foundational data for in-depth analysis to uncover additional toxicity indicators such as alanine aminotransferase and aspartate aminotransferase.

In this study, we conducted comprehensive gene expression and functional characterization of well-differentiated liver organoids, confirming their potential as a predictive model for hepatic toxicity. We propose them as a promising model capable of offering a more physiologically relevant model compared to traditional 2D cultures. Further advancements in organoid technology would enhance our understanding of drug-induced liver injury and facilitate the development of safer pharmaceuticals. Continued research efforts will aim to refine organoid models and validate them as reliable toxicity models for testing various compounds, not only pharmaceuticals but also food substances and chronic toxic substances.

## 5. Conclusions

In this study, hepatic organoids were differentiated and matured to evaluate key liver functions and assess differences in gene expression. Utilizing these hepatic organoids, toxicity evaluations were conducted, examining not only cell viability but also several promising toxicity endpoints. Continued development of such organoid models for toxicity prediction would render them highly valuable. However, further research is needed to refine liver organoids to better mimic the human liver for the comprehensive toxicity evaluation of various substances, extending beyond pharmaceuticals.

## Figures and Tables

**Table 1:** Composition of hepatic media.

Reagent	Company	Catalog Number	Concentration
Advanced DMEM/F12	Thermo Fisher Scientific ^1^	12634028	1×
Penicillin Streptomycin	Thermo Fisher Scientific ^1^	15140122	1%
GlutaMAX	Thermo Fisher Scientific ^1^	35050079	1%
HEPES (1 M)	Thermo Fisher Scientific ^1^	15630080	10 mM
N2 supplement (100×)	Thermo Fisher Scientific ^1^	17502048	1×
N-Acetylcysteine	Sigma-Aldrich ^2^	A9165	1 mM
[Leu15]-Gastrin I human	Sigma-Aldrich ^2^	G9145	10 nM
Recombinant human EGF	PeproTech ^3^	AF-100-15	50 ng/mL
Recombinant human HGF	PeproTech ^3^	100-39	25 ng/mL
B27 supplement without Vit A	Thermo Fisher Scientific ^1^	12587010	1×
A83-01	Tocris Bioscience ^4^	2939	5 μM
Nicotinamide	Sigma-Aldrich ^2^	N0636	10 mM
Forskolin	Sigma-Aldrich ^2^	F3917	10 μM
Recombinant human FGF-basic	PeproTech ^3^	100-18B	10 ng/mL
Oncostatin M	R&D Systems ^5^	295-OM	10 ng/mL
ITS (100×)	Thermo Fisher Scientific ^1^	41400045	5 μg/mL (1×)
Dexamethasone	Sigma-Aldrich ^2^	D4902	100 nM

^1^ Waltham, MA, USA; ^2^ St. Louis, MO, USA; ^3^ Cranbury, NJ, USA; ^4^ Bristol, UK; ^5^ Minneapolis, MN, USA.

**Table 2:** Composition of expansion media.

Reagent	Company	Catalog Number	Concentration
Advanced DMEM/F12	Thermo Fisher Scientific	12634028	1×
Penicillin Streptomycin	Thermo Fisher Scientific	15140122	1%
GlutaMAX	Thermo Fisher Scientific	35050079	1%
HEPES (1 M)	Thermo Fisher Scientific	15630080	10 mM
N2 supplement (100×)	Thermo Fisher Scientific	17502048	1×
N-Acetylcysteine	Sigma-Aldrich	A9165	1 mM
[Leu15]-Gastrin I human	Sigma-Aldrich	G9145	10 nM
Recombinant human EGF	PeproTech	AF-100-15	50 ng/mL
Recombinant human HGF	PeproTech	100-39	25 ng/mL
B27 supplement without Vit A	Thermo Fisher Scientific	12587010	1×
A83-01	Tocris Bioscience	2939	5 μM
Nicotinamide	Sigma-Aldrich	N0636	10 mM
Forskolin	Sigma-Aldrich	F3917	10 μM
Recombinant human R-spondin	R&D Systems	4645-RS-025	1 μg/mL
Recombinant human FGF10	PeproTech	100-26	100 ng/mL
Recombinant human BMP7	PeproTech	120-03P	25 ng/mL

**Table 3:** Composition of differentiation media.

Reagent	Company	Catalog Number	Concentration
Advanced DMEM/F12	Thermo Fisher Scientific	12634028	1×
Penicillin Streptomycin	Thermo Fisher Scientific	15140122	1%
GlutaMAX	Thermo Fisher Scientific	35050079	1%
HEPES (1 M)	Thermo Fisher Scientific	15630080	10 mM
N2 supplement (100×)	Thermo Fisher Scientific	17502048	1×
N-Acetylcysteine	Sigma-Aldrich	A9165	1 mM
[Leu15]-Gastrin I human	Sigma-Aldrich	G9145	10 nM
Recombinant human EGF	PeproTech	AF-100-15	50 ng/mL
Recombinant human HGF	PeproTech	100-39	25 ng/mL
B27 supplement w Vit A	Thermo Fisher Scientific	17504044	1×
A83-01	Tocris Bioscience	2939	0.5 μM
Dexamethasone	Sigma-Aldrich	D4902	3 μM
DAPT	Sigma-Aldrich	D5942	10 μM
Recombinant human BMP7	PeproTech	120-03P	25 ng/mL
Recombinant human FGF19	PeproTech	100-32	100 ng/mL

**Table 4:** List of RT-PCR oligo primer sequences.

Name	Sequence (5′ → 3′, Forward)	Sequence (5′ → 3′, Reverse)
ALB	TTTATGCCCCGGAACTCCTTT	AGTCTCTGTTTGGCAGACGAA
HNF4α	GGCCAAGTACATCCCAGCTTT	CAGCACCAGCTCGTCAAGG
CYP3A4	CTTCATCCAATGGACTGCATAAAT	TCCCAAGTATAACACTCTACACAGACAA
TTR	TGGGAGCCATTTGCCTCTG	AGCCGTGGTGGAATAGGAGTA
β-actin	GGACTTCGAGCAAGAGATGG	AGCACTGTGTTGGCGTACAG

**Table 5:** The list of drugs.

Drug Name	Manufacturer	Catalog Number
Ketoconazole	Sigma-Aldrich	K1003
Tolcapone	Sigma-Aldrich	SML0150
Troglitazone	Toronto Research Chemical Inc.	T892500
Sucrose	Sigma-Aldrich	S9378
Ascorbic acid	Sigma-Aldrich	A4403
Biotin	Sigma-Aldrich	B4639

## Data Availability

The original contributions presented in the study are included in the article, and further inquiries can be directed to the corresponding authors.

## Supplementary Materials

The following supporting information can be downloaded at: https://www.mdpi.com/article/10.3390/toxics12060371/s1, Table S1. List of RT-PCR oligo primer sequences. Figure S1. RT-qPCR of long term differentiated liver organoids and primary human hepatocyte. mRNA expression of hepatocyte markers (A), phase I enzymes of xenobiotics (B), and drug transporter (C). Data are expressed as mean ± standard deviation (n = 3) and compared by one-way analysis of variance with Tukey’s post-hoc to HM. * *p* < 0.05; ** *p* < 0.01; and *** *p* < 0.001.
