# Supplementary material for: Validating Well-Functioning Hepatic Organoids for Toxicity Evaluation
Source: Toxics. 2024 May 17;12(5):371. doi: 10.3390/toxics12050371 (PMC11126009; doi:10.3390/toxics12050371)
Supplement: Supplementary file 1 [file toxics-12-00371-s001.zip › toxics-2928926-supplementary.pdf]

Table S1. List of RT-PCR oligo primer sequences.

Name	Sequence (5′ → 3′, forward)	Sequence (5′ → 3′, reverse)
CYP2D6	TGA AGG ATG AGG CCG TCT GGG AGA	CAG TGG GCA CCG AGA AGC TGA AGT
CYP2C8	CAT TAC TGA CTT CCG TGC TAC AT	CTC CTG CAC AAA TTC GTT TTC C
CYP2C9	CAG AGA CGA CAA GCA CAA CCC T	GTG GCT CCT GTC TTG CAT GCA
CYP2C19	CTG TGC CTG GTC TAA TGT TAC TT	AAG CAT GAC AAA ACA CAT GAG C
MDR1	TTG CTG CTT ACA TTC AGG TTT CA	AGC CTA TCT CCT GTC GCA TTA
MRP2	TGC AGC CTC CAT AAC CAT GAG	GAT GCC TGC CAT TGG ACC TA
NTCP	TGA TGC TCT TCC CCA CAT T	CAT GCG CTA TGT CAT CAA GG
β-actin	GGACTTCGAGCAAGAGATGG	AGCACTGTGTTGGCGTACAG

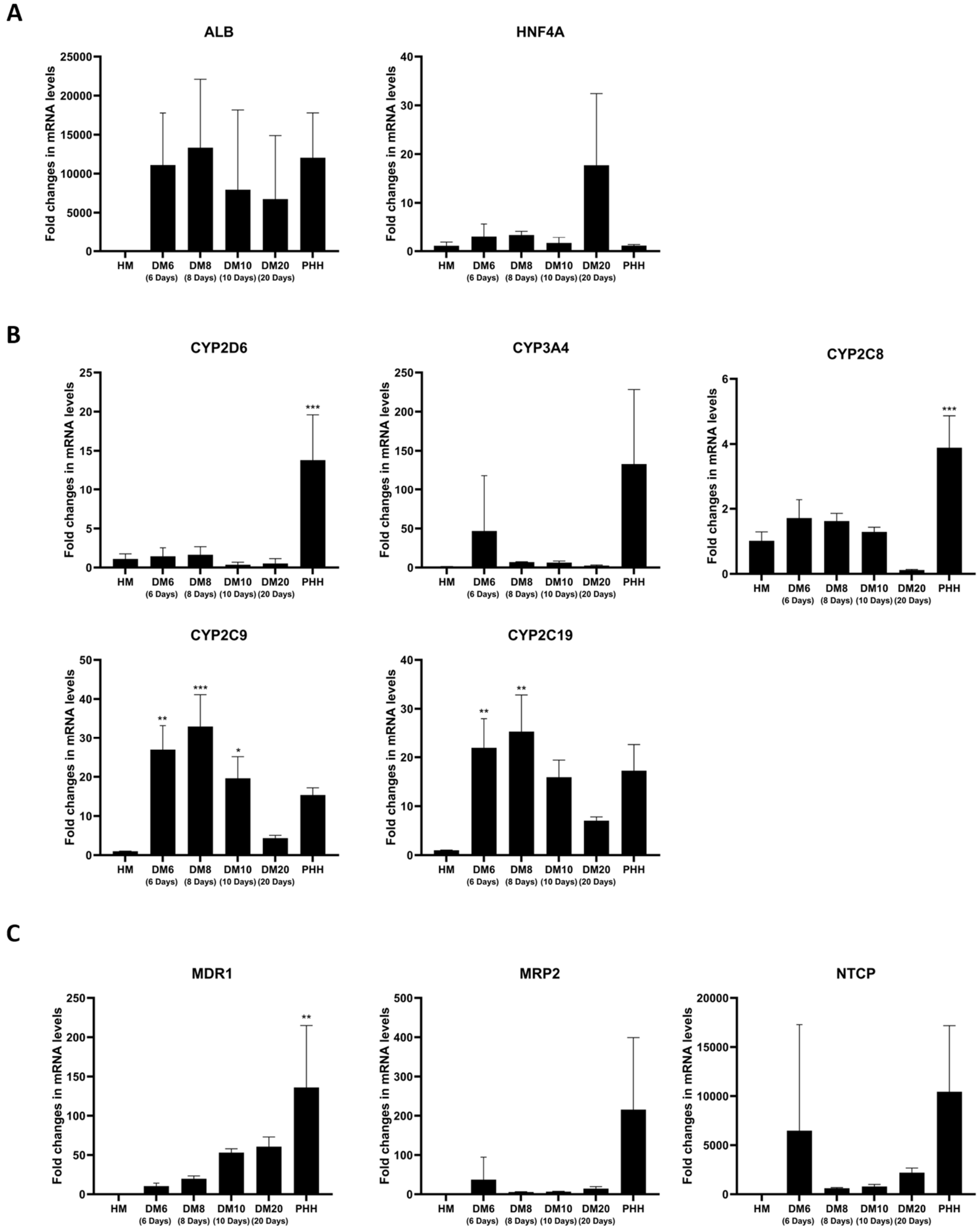

Figure S1. RT-qPCR of long term differentiated liver organoids and primary human hepatocyte. mRNA expression of hepatocyte markers (A), phase I enzymes of xenobiotics (B), and drug transporter (C). Data are expressed as mean ± standard deviation (n = 3) and compared by one-way analysis of variance with Tukey's post-hoc to HM. * $p < 0.05$; ** $p < 0.01$; and *** $p < 0.001$.
